# Cross-Attention and Deep Supervision UNet for Lesion Segmentation of Chronic Stroke

**DOI:** 10.3389/fnins.2022.836412

**Published:** 2022-03-22

**Authors:** Manjin Sheng, Wenjie Xu, Jane Yang, Zhongjie Chen

**Affiliations:** ^1^School of Informatics, Xiamen University, Xiamen, China; ^2^Department of Cognitive Science, University of California, San Diego, San Diego, CA, United States; ^3^Department of Neurology, Zhongshan Hospital, Xiamen University, Xiamen, China

**Keywords:** lesion segmentation, chronic stroke, deep learning, MRI, ATLAS

## Abstract

Stroke is an acute cerebrovascular disease with high incidence, high mortality, and high disability rate. Determining the location and volume of the disease in MR images promotes accurate stroke diagnosis and surgical planning. Therefore, the automatic recognition and segmentation of stroke lesions has important clinical significance for large-scale stroke imaging analysis. There are some problems in the segmentation of stroke lesions, such as imbalance of the front and back scenes, uncertainty of position, and unclear boundary. To meet this challenge, this paper proposes a cross-attention and deep supervision UNet (CADS-UNet) to segment chronic stroke lesions from T1-weighted MR images. Specifically, we propose a cross-spatial attention module, which is different from the usual self-attention module. The location information interactively selects encode features and decode features to enrich the lost spatial focus. At the same time, the channel attention mechanism is used to screen the channel characteristics. Finally, combined with deep supervision and mixed loss, the model is supervised more accurately. We compared and verified the model on the authoritative open dataset “Anatomical Tracings of Lesions After Stroke” (Atlas), which fully proved the effectiveness of our model.

## Introduction

According to the definition of the World Health Organization, stroke is a sudden condition caused by blood vessels in the brain. The brain is the overall control center of various activities of the human body so stroke can lead to disability or death. The incidence rate of stroke has been increasing in recent years. Therefore, the study of cerebral apoplexy has become a popular direction of clinical medicine. In the past, stroke has become a leading cause of death and the second leading cause of disability after ischemic heart disease (Feigin, 2019). With the development of society, due to the changes in the living environment and corresponding lifestyle changes, the number of stroke patients is increasing daily. Hypertension, poor diet, poor physical activity habits, diabetes, obesity, dyslipidemia, as well as psychosocial stress, economic status, air pollution, and other factors, are risk factors for stroke (Pandian et al., 2018). These more common factors have made stroke a common disease with an increasing number of patients. The study of Feigin, VL (Feigin, 2019) showed that the morbidity and mortality of stroke victims had increased significantly since 2010, and the number of strokes in 2016 was twice that in the 1990s.

Stroke is mainly divided into ischemic stroke and hemorrhagic stroke, which are caused by vascular obstruction and vascular rupture, respectively. Once cerebral apoplexy occurs, it may cause permanent damage to the brain and lead to a variety of complications, which can easily lead to disability or even death of patients. Patients who recover are also prone to relapse, therefore, stroke not only affects the normal life of patients, but even endangers the life and health of patients. In addition, the occurrence of stroke will also bring a certain burden to the family and society. Therefore, timely and effective detection and identification of the location of the disease is the basis of minimizing injury. With the development of medical imaging technology, the clinical treatment and intervention of stroke patients has become convenient, accurate, and efficient through the assistance of medical imaging. The identification and analysis of disease location of cerebral apoplexy by medical imaging are now the main diagnostic method in clinical practice. There are three imaging methods for damaged brain tissue of stroke patients: CT angiography (CTA), magnetic resonance imaging (MRI), and digital subtraction angiography (DSA). CT imaging performed well in the diagnosis of tumor-induced stroke patients, but the imaging results of brain regions other than the brain and early lesions were weak. Meanwhile, angiography requires the injection of a contrast agent into the patient, which has implications for the patient’s body. Non-invasive brain MRI results, by contrast, are not only for ischemic stroke imaging but is more sensitive, and can accurately analyze the lesions. In addition, for hemorrhagic stroke in the different periods of the hemorrhage of magnetic resonance imaging results, through multiple check sequences, it can realize direct imaging of multidimensional no dead angle, which provides favorable conditions for the doctor’s clinical diagnosis and treatment. Therefore, this paper focuses on T1-Weighted Magnetic Resonance Images of cerebral apoplexy patients to study the location segmentation of lesions in the images.

At present, the number of studies on the segmentation of acute stroke is much larger than that on the segmentation of chronic stroke, and the study of chronic stroke is of great significance for early intervention treatment and prevention of serious consequences. According to whether manual participation is needed in the algorithm, the segmentation methods of stroke lesions can be divided into semi-automatic and automatic categories at present. Clustering is the most widely used semi-automatic segmentation method. Haan et al. (2015) segmented through the iterative region growing based on local intensity maximum. However, this method not only requires a certain amount of manpower because of the need for artificial selection of clusters, but also its effect is greatly influenced by human experience. In automatic segmentation, machine learning is also a mainstream method. For example, Seghier et al. (2008) used fuzzy means clustering to identify the location of brain lesions. However, the application scope of this method is limited, and it is only applicable to single types of images. The sensitivity of this method is limited by parameters and other factors. Griffis et al. (2016) classified voxels by Gaussian Naive Bayes, but this method is prone to miss detection for small lesion areas. Pustina et al. (2016) used the random forest algorithm and combined it with adjacent voxels for analysis, but this method had the defect of high computational cost. Meanwhile, Ito et al. (2019) conducted an experimental comparison of the above three methods, and the results showed that these methods had limitations in the segmentation of lesions in the cerebellum and brain stem.

In addition, the experiment of Maier et al. (2015) also showed that machine learning had a good performance in the segmentation task of stroke lesions. In machine learning methods, in addition to model-based Gaussian Naive Bayes, random forest, and k-neighborhood algorithm, there are also deep learning methods taking convolutional neural network (CNN) as an example. Deep learning methods have many mature and robust methods in the field of medical image classification (Lyu et al., 2021; Zhu et al., 2021a,b). At the same time, there has been much development in the field of medical image segmentation. Among them, CNN and full convolutional neural network (FCN) show obvious advantages in the segmentation of lesions in brain images (Karthik et al., 2020). Lucas et al. (2017) applied the classical fully connected neural network to the segmentation of stroke lesions. In addition, U-Net architecture also shows a good effect in image segmentation, so there are many applications of FCN, U-Net, and their variants in the segmentation task of stroke lesions. Liu et al. (2018) proposed a fully convolutional network with a residual structure (Res-FCN). Hui et al. (2020) combined the attention mechanism with U-Net and improved it to increase the segmentation accuracy by suppressing the irrelevant regions of lesions through the attention generated by multi-scale features. Dolz et al., 2019) obtained more modal information through individual processing of the input image and extended the convolution block of InceptionNet to obtain more context information to improve performance. However, U-Net architecture is limited by the size of convolutional features, and the simple stacking of features during decoding limits the diversity of information. Therefore, Qi et al. (2019) proposed an X-Net based on deep separable convolution, which designed a non-local operation, namely feature similarity module (FSM), to capture remote dependencies. Using deep convolution can reduce the scale of the network, and the developed FSM can provide more effective and dense context information extraction, which is conducive to better segmentation.

Unfortunately, these methods often ignore the size and location information of lesions, do not make full use of the extracted feature information, and lose the early useful features. Therefore, we propose an end-to-end model named cross-attention and deep supervision UNet (CADS-UNet), which refers to the classical UNet structure, designs the cross-space attention module (CSAM) and the channel attention module (CAM), filters the features of each layer, and extracts more valuable features. In summary, the contributions of this paper are summarized as follows.

(1) We propose CADS-UNet to effectively segment the lesion region of stroke. Through the cross-spatial attention module and CAM, important information is enhanced and the effectiveness of features is improved.

(2) We use a mixed loss function and deep supervision to improve the efficiency of network training and provide strong normalization through supervising the middle layer.

(3) We conducted comparative experiments and ablation experiments on the Atlas dataset and compared with advanced methods to show the advantages.

## Materials and Methods

**FIGURE 1:**
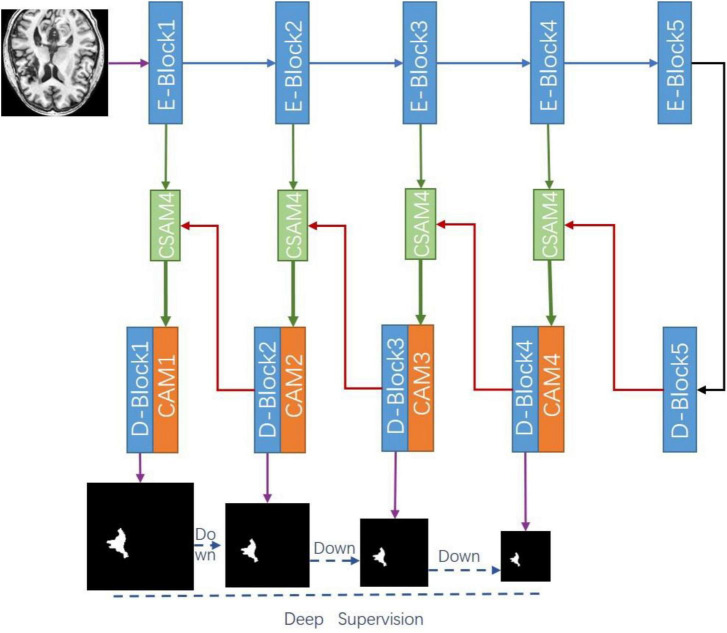
Overview of our proposed cross-attention and deep supervision UNet (CADS-UNet).

Figure 1 shows the overall architecture of our proposed CADS-UNet. On the general encoder-decoder architecture, we add the cross spatial attention module and CAM to effectively select features, fully mix features from different levels, and guide segmentation. We utilize ResNet34 (He et al., 2016) as the encoder, which contains a total of five blocks. Each decoder block consists of two Conv-BN-ReLU combinations. At the same time, the deep supervision algorithm is combined to improve the directness and transparency of the hidden layer learning process. In this section, each module of our network will be analyzed in detail. In section “Cross-Spatial Attention Module,” we introduce the cross spatial attention module (CSAM), the CAM in section “Channel Attention Module,” and finally, in section “Deep Supervision and Mixing Loss Function,” we introduce the deep supervision and mixing loss function.

### Cross-Spatial Attention Module

Convolutional neural network extracts the features of the target by layer abstraction and one of the important concepts is a receptive field. The receptive field of a low-level network is relatively small and the representational ability of geometric detail information is strong. It tends to extract low-level features such as the edgy texture of an image. Although the resolution of low-level features is high, the representational ability of semantic information is weak. With the increase of neural network layers, the receptive field increases, and the representational ability of semantic information is strong, but the resolution of the feature map is low and the representational ability of geometric information is weak. The classical U-Net performs up-sampling four times and uses skip connection in the same stage to combine the encoded feature map with the decoded feature map so that the feature map integrates more low-level features and the features of different scales. Due to the spatial uncertainty of stroke lesions, to obtain more effective spatial features, we propose a CSAM to replace the skip connection in U-Net. This module extracts a wide range of location-sensitive information at encoding and decoding layers, respectively, and cross-encodes them into feature map.

CSAM is a soft spatial attention strategy (Tao et al., 2018) that selects fine-grained important pixels which are pixel level. The purpose is to combine features with spatial location strategy, so that the obtained features pay more attention to uncertain and complex regions, to realize hierarchical feature complimentary and prediction refinement. Different from the common self-attention module (Park et al., 2018), we select features by cross-weighting the spatial features of the encoding layer and decoding layer. The features of the coding layer include unique edge space features, while the features of the decoding layer include high-level abstract space features. Both sides focus on the features of different levels and lose the spatial focus of the other layer. Directly connecting encoding and decoding features cannot efficiently select spatial information. Therefore, we use the soft attention mechanism to cross weight the features, so that the features are concentrated in different key parts of space to prevent the loss of effective information and strengthen important information.

**FIGURE 2:**
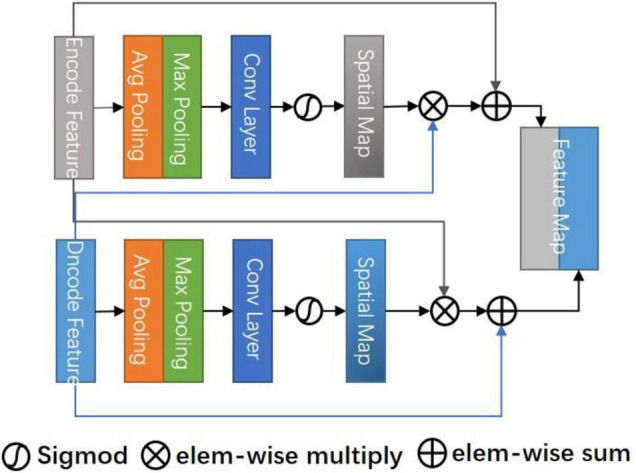
Cross space attention module (CSAM).

As shown in Figure 2, the attention diagram of each CSAM module is generated by the decoder and encoder of the corresponding layer. The input feature maps of CSAM are the encoding feature *F*_*e*_ ∈ ℝ^*C*×*H*×*W*^ and the decoding feature *F*_*d*_ ∈ ℝ^*C*×*H*×*W*^, in which *C* is the number of channels and *H*, *W* are the height and width of the feature map, respectively. To compute the spatial attention, first applying max pooling and avg pooling operations along the channel axis to obtain two 2D maps, then concatenate them according and send the concatenated feature descriptor to a standard convolution layer. After activating function, the spatial attention map is obtained, and its formula is expressed as:


$$\text{M}\left(F\right)=\sigma \left({\text{f}}^{3\times 3}\left(\left[\text{AvgPool}\left(F\right);\text{MaxPool}\left(F\right)\right]\right)\right)$$

Where **σ** represents the sigmoid function and ***f*^3×3^** denotes a convolution operation with the filter size of **3**×**3**.

The spatial attention maps of *F_e_* and *F*_*d*_ are extracted, respectively, and the original features are weighted by cross-multiplication to obtain a hybrid spatial selection feature map. Finally, the original features are summed as residual blocks to avoid overfitting. The specific formula is as follows:


$${\text{S}}_{S}\left({\text{F}}_{e},{\text{F}}_{d}\right)=\left[\left(\text{M}{\text{F}}_{e}⊗{\text{F}}_{d}+{\text{F}}_{d}\right);\left(\text{M}{\text{F}}_{d}⊗{\text{F}}_{e}+{\text{F}}_{e}\right)\right]$$

Where ⊗ denotes element-wise multiplication, it can be inferred from the formula that the final result map is the sum of relational features and original features. Therefore, *S_s_* has a wide range of contextual perspectives, effectively aggregates spatial information through the cross-spatial attention module, provides local context enhancement of different receptive fields for each position feature column of each decoding layer, and skillfully uses the prediction confidence of the decoding layer as a guide to force the current layer to focus on difficult.

### Channel Attention Module

**FIGURE 3:**
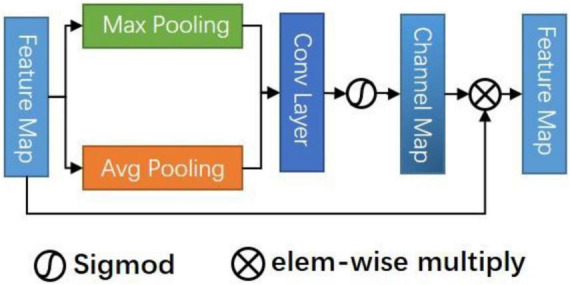
Channel attention module (CAM).

Figure 3 shows the CAM. In a sense, the channel of a neural network is the feature, and the number of channels is the feature number. In addition to spatial location information, channel information is also an important information source for selecting effective feature maps. The mixed attention mechanism is better than the single attention mechanism (Woo et al., 2018). So, we use the CAM serially after the CSAM to select the combined multi-feature channels. We hope to improve the representation ability of the network modeling the dependence of each channel and adjusting the features channel by channel, in this way, the network can learn to selectively strengthen the features containing useful information and suppress useless channel features globally.

Specifically, CAM takes the connected mixed features *S*_*s*_ *F*_*e*_, *F*_*d*_ as inputs to suppress or enhance the channel strength by modeling the importance of each channel. Firstly, max pooling and avg pooling are used to summarize the spatial information about the feature graph. We obtain two descriptors and then forward them to a shared multi-layer perceptron (MLP) that contains two layers of FC, respectively. Finally, the pixels are added and fused, and the channel attention map is obtained through an activation function. The formula is:


$$\text{M}\left({\text{S}}_{s}\right)=\sigma \left(\text{MLP}\left(\text{AvgPool}\left({\text{S}}_{s}\right)\right)+\text{MLP}\left(\text{MaxPool}\left({\text{S}}_{s}\right)\right)\right)$$

By multiplying the original feature mapping with the attention value, we can identify some informative contextual features and suppress those features that are not conducive to improving the discrimination.


$${\text{S}}_{c}\left({\text{S}}_{s}\right)=\text{M}{\text{S}}_{s}⊗{\text{S}}_{s}$$

### Deep Supervision and Mixing Loss Function

We add cross-space and channel dual attention modules in each decoding layer. In addition, we use deep supervision (Lee et al., 2014) to introduce intermediate loss supervision functions for each hidden layer to improve the directness and transparency of the hidden layer learning process. These intermediate loss functions can be regarded as additional (soft) constraints on the learning process. At the same time, it can solve the problems such as the disappearance of training gradient and slow convergence speed of deep neural networks.

In the training process, deep supervision may lead to lazy convergence of the deep network. Therefore, we propose a new loss function, which adopts different loss functions according to the output of each layer and combines them.

#### Binary Cross Entropy Loss

For the segmentation problem of front and back scene pixel-level classification, the most widely used objective function is the binary cross-entropy (BCE) loss, which is defined as follows:


$${\text{L}}_{\mathrm{BCE}}=\frac{1}{\text{N}}\sum_{i=1}^{N}{\text{y}}_{i}\text{log}{\hat{\text{y}}}_{i}+\left(1-{\text{y}}_{i}\right)\text{log}\left(1-{\hat{\text{y}}}_{i}\right)$$

Where *N* is the total number of pixels, *y_i_* is the real label of pixel *i*, and ${\hat{y}}_{i}$ represents the prediction probability that pixel *i* is classified as “1.” This loss function ignores class imbalance, so the importance of all pixels is the same. In this case, the larger background has more influence on the training process.

#### Dice Coefficient Loss

In 3D medical data, including our MRI stroke data, the number of lesion slices only accounts for a small part of the total number of 3D slices, and the size of lesions is uncertain. In the training process, the number of negative pixels and slices (excluding lesions) is much more than the number of positive pixels, and a large number of negative regions may dominate the loss function. Therefore, using BCE loss alone cannot meet our task. Dice coefficient loss (DL) (Milletari et al., 2016) does not take into account the real negative pixels, alleviates the imbalance between background and foreground pixels, and can achieve better results in unbalanced segmentation. Sim ilar to Dice score, dice loss is defined as follows:


$${\text{L}}_{\mathrm{Dice}}=1-\frac{2\sum_{i}{\text{y}}_{i}{\hat{\text{y}}}_{i}+\epsilon }{\sum_{i}{\hat{\text{y}}}_{i}+\sum_{i}{\text{y}}_{i}+\epsilon }$$

Where ϵ∈[0, 1] is a tunable parameter to prevent a divide-by-zero error and make negative samples also have gradient propagation.

#### Mixed Loss

Based on the above two losses, we propose a mixed loss (ML) to improve the convergence. Combined with the deep supervision strategy, we use the dice coefficient loss function for the coarser step 3 and 4 outputs, and *L*_*BCE*_ + *L*_*Dice*_ loss function for the finer step 1 and 2 outputs. For different strides, different loss functions are used, because the coarse output only focuses on the global structure and ignores the local details, while the fine output achieves pixel-level accuracy by relying on local clues, and we use α,β to add weights for different layers. Finally, the mixed loss is as follows:


$$\text{L}=\left({\text{L}}_{\mathrm{Dice}}^{1}+{\text{L}}_{\mathrm{BCE}}^{1}\right)+\alpha \left({\text{L}}_{\mathrm{Dice}}^{2}+{\text{L}}_{\mathrm{BCE}}^{2}\right)+\beta \left({\text{L}}_{\mathrm{Dice}}^{3}+{\text{L}}_{\mathrm{Dice}}^{4}\right)$$

## Experiments and Results

### Datasets

To evaluate the performance of the proposed method in the segmentation of stroke lesions, we used a subset of the open data set “Anatomical Tracings of Lesions After Stroke” (Atlas) (Liew et al., 2018). Atlas dataset consists of 304 MRI images from 11 cohorts around the world. A total of 11 experts who had received standardized training in lesion recognition and segmentation manually drew lesion masks for the data set. Due to the difference in technical difficulty and scanner image quality, 229 brain data were converted into standard MNI space. Therefore, experiments are only carried out on these 229-subset data.

All T1 weighted MRI data are collected on a 3T MRI scanner with a resolution of 1*mm*^3^(isotropic). The mean lesion volume of all cohorts is 2.128 ± 3.898×10^4^mm^3^. The minimum lesion size is 10*mm*^3^ and the maximum lesion size is 2.838×10^5^mm^3^. On average, a single patient is more likely to have only one lesion (58%). In the left hemisphere, right hemisphere, and other parts (such as the brain stem), the probability of identifying lesions is roughly the same (48.4, 43.8, and 7.7%, respectively). Overall, the number of subcortical lesions (70.7%) is higher than that of cortical lesions (21.5%).

The experimental data consists of 229 T1 weighted standardized 3D MR images, in which each 3D MR image consists of 189 2D slices, and the size of each slice is 233 × 197. The 2D segmentation method was used in this experiment, so the slices are standardized. The data set contains 43281 slices. We randomly selected 137 cases for training, 36 cases for verification, and 56 cases for testing.

### Implementation Details

In data preprocessing, each case was cut into a cross-sectional image. Each image was cropped using diagonal coordinates (10,40) and (190,220). The cropped region removed irrelevant information and expanded the proportion of stroke lesions in the whole image. Next, the size of the cropped image was adjusted to 192 × 192 using bilinear interpolation, and saved each slice in.npy format for later use. The training data were shuffled randomly and all slice masks were cut to the same size.

In this experiment, Adam (Kingma and Ba, 2014) optimizer was used to adjust the initial learning rate and the initial learning rate was set to *init*_*lr* = 1*e*^−3^. We adjusted the initial learning rate by using multi learning rate strategy, which was $lr=init\_lr\times {\left(1-\frac{epoch}{nEpoch}\right)}^{power}$, where *power* = 0.9, *nEpoch* = 100. Due to the memory limitation of GPU, the batch size was set to 24. Our model was implemented using the PyTorch (Paszke et al., 2019) framework.

We selected a series of evaluation indicators to quantify the performance of the proposed model. We calculated evaluation scores for each patient in the test set on 3D MRI (189 2D slices) and reported the mean Dice Similarity Coefficient (DSC), Precision, and Recall. In addition, we also tested the global DSC of all data. DSC is the main indicator to evaluate the difference between the model prediction and the ground truth, which is used to quantify the overlap ratio between the two masks. Precision, also known as the positive predictive value, is the ratio of true positive to the overall predictive result. The recall is an integrity measure defined as the ratio of true positive to the entire basic fact. They are defined as:


$$\text{DSC}=\frac{2\text{TP}}{2\text{TP}+\text{FP}+\text{FN}}$$

$$\text{Recall}=\frac{\text{TP}}{\text{TP}+\text{FN}}$$

$$\text{Precision}=\frac{\text{TP}}{\text{TP}+\text{FP}}$$

We threshold all predicted results. We set it to 0 when the probability of a pixel being predicted to be foreground is less than 0.5, and 1 otherwise. It is positive when the pixel predicted value is the same as the true value, otherwise it is negative. Where true positive (TP) indicates that the model correctly predicted pixel. False-positive (FP) indicates the pixel that the model misclassifies to be positive. False-negative (FN) indicates that the positive pixel is mistakenly classified to be negative by the model.

### Comparison of Different Methods

To evaluate the performance of our algorithm, we selected four models for experimental comparison (Table 1), namely FCN-8s (Long et al., 2015), U-Net (Ronneberger et al., 2015), ResUNet (Zhang et al., 2018), and Attention-UNet (Oktay et al., 2018). As the baseline of the segmentation network, the U-Net network has a simple and clear structure and has a good effect. Subsequent networks, including our network, are improved based on U-Net. ResUNet combines the idea of ResNet and adds a residual module to each block. Attention-UNet monitors the features of the upper level through the features of the lower level to realize the attention mechanism. All network parameters are configured according to section “Implementation Details.” These networks are also implemented on a unified platform. The training set and verification sets are strictly consistent (including data preprocessing). During training, the batch size is set to 24 and the loss is set to *L*_*Dice*_ + *L*_*BCE*_.

**TABLE 1:** Comparison with state-of-the-art methods on the ATLAS dataset.

Method	DSC	DSC (global)	Recall	Precision
FCN-8s	0.4274	0.6720	0.4531	0.4883
U-Net	0.4944	0.6933	0.5030	0.6419
ResUNet	0.5081	0.7373	0.5081	0.5921
Attention-UNet	0.5162	0.7383	0.5321	**0.6518**
CADS-UNet (ours)	**0.5564**	**0.7451**	**0.5817**	0.6368

*Bold value shows the best performance.*

By analyzing the results in Table 1, we can see that the effect of classical FCN-8s is the worst, only 0.4274 (DSC). Taking the result of U-Net 0.4944 (DSC) as the dividing line, the results of ResUNet and Attention-UNet are improved. The best result of Attention-UNet is 0.5162 (DSC), which also proves the usefulness of the attention mechanism. Our model obtained a DSC of 0.5564, which scored the highest among all models, and several other indicators [DSC (global), Recall, Precision] were 0.7451, 0.5817, and 0.6368, respectively. Therefore, from the experimental point of view, our model is optimal.

**FIGURE 4:**
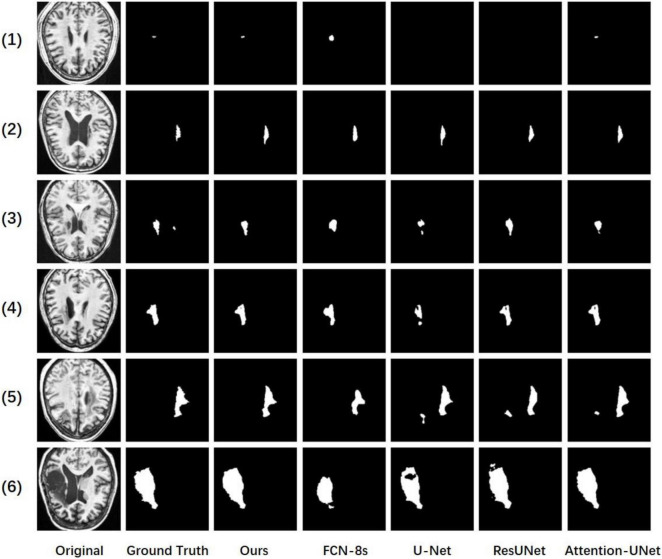
Comparisons of our method, baseline, FCN-8s, U-Net, ResUNet, and attention-UNet.

Figure 4 shows some of our cases. We arrange the images from small to large according to the size of the lesion. Where the second column is the ground truth and our resulting graph is in the third column. In the first row, we can see that for very small lesion areas, U-Net and ResUNet cannot segment the lesion, the predicted mask is empty, and the segmentation result of FCN-8s is not good. In contrast, our model and Attention-UNet can accurately segment the lesion area. Therefore, compared with other models, our method has advantages in identifying and segmenting small lesion samples. In addition, take the result in line 5 as an example, U-Net, ResUNet, and Attention-UNet mistakenly recognized the tissue with the low-intensity signal as the lesion area of stroke and the segmentation is not accurate enough. Our model does not segment the non-lesion area and the segmentation shape is most similar to the ground truth. Other results also show that our model obtains smoother edge results and a more accurate segmentation rate. The experimental results show that the proposed CADS-UNet has strong robustness and effectiveness.

### Comparison With Other Methods and Ablation Experiments

Table 2 compares our model (CADS-UNet) with other existing methods on the same data set. All comparison results were obtained directly from the paper. We did not reproduce the experiment because we believe in the best results reported by other authors. We selected five methods based on deep learning (X-Net, 2D MI-UNet, 2.5D CNN, D-UNet, and 3D-UNet). The results of X-Net and D-UNet were obtained from Qi et al. (2019) and Zhou et al. (2019). The results of 2.5D CNN were obtained from Xue et al. (2020) and the results of 3D-UNet and 2D MI-UNet were reported by Zhang et al. (2020). As can be seen from the Table, compared with other methods, our model also has certain advantages.

**TABLE 2:** Comparison with other state-of-the-art methods and ablation studies on the ATLAS dataset.

Method	DSC	Recall	Precision	Train/Test
X-Net	0.4867	0.4752	0.6000	All/Fivefold cross-validation
2D MI-UNet	0.4945	0.5237	0.5669	All/Fivefold cross-validation
3D UNet	0.5296	0.5497	0.6090	All/Fivefold cross-validation
D-UNet	0.5349	0.5243	0.6331	183/46
2.5D CNN	0.54	–	–	99/Fivefold cross-validation
CADS-UNet (ours)	**0.5564**	**0.5817**	0.6368	137/56
BaseLine (BL)	0.5124	0.5291	0.6111	137/56
BL + CSAM	0.5361	0.5455	**0.6451**	137/56
BL + CSAM + CAM	0.5407	0.5654	0.6218	137/56

*Bold value shows the best performance.*

In order to verify the necessity and effectiveness of each module (CSAM, CAM, DL), we conducted ablation experiments between different modules. Specifically, the baseline model refers to the encode-decode network, the feature connection adopts connection instead of direct addition to double the number of channels. Before adding the deep supervision module, our loss adopts *L*_*Dice*_ + *L*_*BCE*_. We gradually add CSAM, CAM and DL modules to the base model, which are expressed in the table as baseline, BL + CSAM, BL + CSAM + CAM, and ours (BL + CSAM + CAM + DL). From the results, we can see that with the introduction of the module, the effect of our algorithm has been gradually improved, boosting dice by 2.37, 0.46, and 1.57%, respectively. The addition of the CSAM module greatly improves the accuracy, which also proves that the model does capture the corresponding spatial location information, and the joint use of CAM and CSAM also improves the model. Finally, a specific loss function constrains the output of each layer, making the focus of the attention mechanism more accurate, so that the whole model reaches the optimum. The experimental results show that our added module is indeed effective.

## Conclusion

We propose a new method to realize the automatic segmentation of stroke lesions. The network strengthens the feature space information through the cross-spatial attention module so that the model pays more attention to uncertain and complex regions. At the same time, the channel information is filtered through the CAM, the effective channel information is strengthened, and more accurate feature information is captured. Finally, the deep supervision strategy combined with the mixed loss function solves the problems of the disappearance of the training gradient and the slow convergence speed of the deep neural network. The model is evaluated on the famous open data set ATLAS, which proves the superiority of our model. The proposed model has a clear structure and robustness. In the future, we plan to validate our method on more clinical datasets and improve the model in combination with 3D convolutions to further improve the performance of the model. Due to the continuity of the lesion block, 3D convolution can add spatial information to the model, which can theoretically further improve our segmentation accuracy, but 3D convolution has higher requirements for computing performance. The 2.5D network that combines 2D and 3D is also our future research direction.

## Data Availability Statement

The datasets analyzed for this study can be found in the ATLAS dataset: http://fcon_1000.projects.nitrc.org/indi/retro/atlas_download.html.

## Ethics Statement

Ethical review and approval was not required for the study on human participants in accordance with the local legislation and institutional requirements. The patients/participants provided their written informed consent to participate in this study. Written informed consent was obtained from the individual(s) for the publication of any potentially identifiable images or data included in this article.

## Author Contributions

MS wrote the main manuscript and conducted the experiments. WX and JY participated in the writing of the manuscript and modified the English grammar of the manuscript. JY and ZC contributed to the implementation of the method. All authors contributed to the revision, proofreading, and revision of the manuscript.

## Conflict of Interest

The authors declare that the research was conducted in the absence of any commercial or financial relationships that could be construed as a potential conflict of interest.

## Publisher’s Note

All claims expressed in this article are solely those of the authors and do not necessarily represent those of their affiliated organizations, or those of the publisher, the editors and the reviewers. Any product that may be evaluated in this article, or claim that may be made by its manufacturer, is not guaranteed or endorsed by the publisher.

## References

[B2] Dolz J. Ayed I. B. Desrosiers C. (2019). “Dense multi-path U-net for ischemic stroke lesion segmentation in multiple image modalities,” in *Brainlesion: Glioma, Multiple Sclerosis, Stroke and Traumatic Brain Injuries, Brainles 2018*, Vol. 11383 eds Crimi A. Bakas S. Kuijf H. Keyvan F. Reyes M. van Walsum T. (Cham: Springer), 271–282. 10.1007/978-3-030-11723-8_27

[B3] Feigin V. L. (2019). Anthology of stroke epidemiology in the 20th and 21st centuries: assessing the past, the present, and envisioning the future. *Int. J. Stroke* 14 223–237. 10.1177/1747493019832996 30794102

[B4] Griffis J. C. Allendorfer J. B. Szaflarski J. P. (2016). Voxel-based Gaussian naive Bayes classification of ischemic stroke lesions in individual T1-weighted MRI scans. *J. Neurosci. Methods* 257 97–108. 10.1016/j.jneumeth.2015.09.019 26432931 PMC4662880

[B5] Haan B. D. Clas P. Juenger H. Wilke M. Karnath H. O. (2015). Fast semi-automated lesion demarcation in stroke. *Neuroimage Clin.* 9 69–74. 10.1016/j.nicl.2015.06.013 26413473 PMC4543214

[B6] He K. Zhang X. Ren S. Sun J. (2016). “Deep residual learning for image recognition,” in *Proceedings of the IEEE Conference on Computer Vision and Pattern Recognition (CVPR)*, (Piscataway, NJ: IEEE), 770–778. 10.1109/CVPR.2016.90

[B7] Hui H. S. Zhang X. Y. Li F. L. Mei X. B. Guo Y. L. (2020). A partitioning-stacking prediction fusion network based on an improved attention U-Net for stroke lesion segmentation. *IEEE Access* 8 47419–47432. 10.1109/ACCESS.2020.2977946

[B8] Ito K. L. Kim H. Liew S. L. (2019). A comparison of automated lesion segmentation approaches for chronic stroke T1-weighted MRI data. *Hum. Brain Mapp.* 40 4669–4685. 10.1002/hbm.24729 31350795 PMC6851560

[B9] Karthik R. Menaka R. Johnson A. Anand S. (2020). Neuroimaging and deep learning for brain stroke detection - A review of recent advancements and future prospects. *Comput. Methods Programs Biomed.* 197:105728. 10.1016/j.cmpb.2020.105728 32882591

[B10] Kingma D. Ba J. (2014). Adam: a method for stochastic optimization. *Comput. Sci.* arXiv [Preprint] arXiv:1412.6980.

[B11] Lee C. Y. Xie S. Gallagher P. Zhang Z. Tu Z. (2014). Deeply-supervised nets. *Proc. Mach. Learn. Res.* 38 562–570. 10.3390/s19092009 31035673 PMC6540294

[B12] Liew S.-L. Anglin J. M. Banks N. W. Sondag M. Ito K. L. Kim H. (2018). A large, open source dataset of stroke anatomical brain images and manual lesion segmentations. *Sci. Data* 5:180011. 10.1038/sdata.2018.11 29461514 PMC5819480

[B13] Liu Z. Y. Cao C. Ding S. X. Liu Z. Han T. Liu S. (2018). Towards clinical diagnosis: automated stroke lesion segmentation on multi-spectral MR image using convolutional neural network. *IEEE Access* 6 57006–57016. 10.1109/access.2018.2872939

[B14] Long J. Shelhamer E. Darrell T. (2015). “Fully convolutional networks for semantic segmentation,” in *Proceedings of the IEEE Transactions on Pattern Analysis and Machine Intelligence*, (Piscataway, NJ: IEEE), 3431–3440. 10.1109/cvpr.2015.7298965 27244717

[B15] Lucas C. Maier O. Heinrich M. P. (2017). “Shallow fully-connected neural networks for ischemic stroke-lesion segmentation in MRI,” in *Bildverarbeitung für die Medizin 2017*, eds Maier-Hein K. geb. Fritzsche Deserno T. geb. Lehmann Handels H. Tolxdorff T. (Berlin: Springer Vieweg), 261–266. 10.1007/978-3-662-54345-0_59

[B16] Lyu Z. Wang Z. Luo F. Shuai J. Huang Y. (2021). Protein secondary structure prediction with a reductive deep learning method. *Front. Bioeng. Biotechnol.* 9:687426. 10.3389/fbioe.2021.687426 34211967 PMC8240957

[B17] Maier O. Schroder C. Forkert N. D. Martinetz T. Handels H. (2015). Classifiers for ischemic stroke lesion segmentation: a comparison study. *PLoS One* 10:e0145118. 10.1371/journal.pone.0145118 26672989 PMC4687679

[B18] Milletari F. Navab N. Ahmadi S.-A. (2016). V-Net: fully convolutional neural networks for volumetric medical image segmentation. *Med. Image Comput. Comput. Assist. Interv.* 9351 234–241. 10.1109/3dv.2016.79

[B19] Oktay O. Schlemper J. Folgoc L. L. Lee M. Heinrich M. Misawa K. (2018). Attention u-net: learning where to look for the pancreas. *arXiv* [preprint]. arXiv:1804.03999,

[B20] Pandian J. D. Gall S. L. Kate M. P. Silva G. S. Akinyemi R. O. Ovbiagele B. I. (2018). Prevention of stroke: a global perspective. *Lancet* 392 1269–1278. 10.1016/S0140-6736(18)31269-8 30319114

[B21] Park J. Woo S. Lee J. Y. Kweon I. S. (2018). “Bam: bottleneck attention module,” in *Proceedings of the British Machine Vision Conference.* arXiv [Preprint]. arXiv 1807.06514.

[B22] Paszke A. Gross S. Massa F. Lerer A. Bradbury J. Chanan G. (2019). PyTorch: an imperative style, high-performance deep learning library. *Adv. Neural Inf. Process. Syst.* 32 8024–8035.

[B23] Pustina D. Coslett H. B. Turkeltaub P. E. Tustison N. Schwartz M. F. Avants B. (2016). Automated segmentation of chronic stroke lesions using LINDA: lesion identification with neighborhood data analysis. *Hum. Brain Mapp.* 37 1405–1421. 10.1002/hbm.23110 26756101 PMC4783237

[B24] Qi K. H. Yang H. Li C. Liu Z. Y. Wang M. Y. Liu Q. G. (2019). “X-Net: brain stroke lesion segmentation based on depthwise separable convolution and long-range dependencies,” in *Proceedings of the Medical Image Computing and Computer Assisted Intervention – MICCAI 2019*, Shenzhen, Vol. 11766 247–255. 10.1007/978-3-030-32248-9_28

[B25] Ronneberger O. Fischer P. Brox T. (2015). U-Net: convolutional networks for biomedical image segmentation. *Med. Image Comput. Comput. Assist. Interv.* 9351 234–241. 10.1007/978-3-319-24574-4_28

[B26] Seghier M. L. Ramlackhansingh A. Crinion J. Leff A. P. Price C. J. (2008). Lesion identification using unified segmentation-normalisation models and fuzzy clustering. *Neuroimage* 41 1253–1266. 10.1016/j.neuroimage.2008.03.028 18482850 PMC2724121

[B27] Tao Y. Z. Sun Q. Du Q. Liu W. (2018). Nonlocal neural networks, nonlocal diffusion and nonlocal modeling. *Adv. Neural Inf. Process. Syst.* 31.

[B28] Woo S. H. Park J. Lee J. Y. Kweon I. S. (2018). “CBAM: convolutional block attention module,” in *Computer Vision – ECCV 2018. Lecture Notes in Computer Science*, Vol. 11211 eds Ferrari V. Hebert M. Sminchisescu C. Weiss Y. (Cham: Springer), 3–19. 10.1007/978-3-030-01234-2_1

[B29] Xue Y. Farhat F. G. Boukrina O. Barrett A. M. Binder J. R. Roshan U. W. (2020). A multi-path 2.5 dimensional convolutional neural network system for segmenting stroke lesions in brain MRI images. *Neuroimage Clin.* 25:102118. 10.1016/j.nicl.2019.102118 31865021 PMC6931186

[B30] Zhang Y. Wu J. Liu Y. Chen Y. Wu E. X. Tang X. (2020). MI-UNet: multi-inputs UNet incorporating brain parcellation for stroke lesion segmentation from T1-weighted magnetic resonance images. *IEEE J. Biomed. Health Inform.* 25 526–535. 10.1109/jbhi.2020.2996783 32750908

[B31] Zhang Z. Liu Q. Wang Y. (2018). Road extraction by deep residual U-Net. *IEEE Geosci. Remote Sens. Lett.* 15 749–753. 10.1109/lgrs.2018.2802944

[B32] Zhou Y. Huang W. Dong P. Xia Y. Wang S. (2019). “D-UNet: a dimension-fusion U shape network for chronic stroke lesion segmentation,” in *Proceedings of the IEEE-ACM Transactions on Computational Biology and Bioinformatics*, Vol. 18 (Piscataway, NJ: IEEE), 940–950. 10.1109/tcbb.2019.2939522 31502985

[B33] Zhu Z. Lu S. Wang S.-H. Gorriz J. M. Zhang Y.-D. (2021a). BCNet: a novel network for blood cell classification. *Front. Cell Dev. Biol.* 9:813996. 10.3389/fcell.2021.813996 35047515 PMC8762289

[B34] Zhu Z. Lv D. Zhang X. Wang S.-H. Zhu G. (2021b). Deep learning in the classification of stage of liver fibrosis in chronic hepatitis b with magnetic resonance ADC images. *Contrast Media Mol. Imaging* 2021:2015780. 10.1155/2021/2015780 35024010 PMC8716233

